# Association of Sarcopenia With Cognitive Function in Patients With Parkinson's Disease: A Cross‐Sectional Study

**DOI:** 10.1002/brb3.71460

**Published:** 2026-04-28

**Authors:** Qiuwan Liu, Kangrui Zhang, Yi Tang, Chi Zhang, Chengjie Mao, Chunfeng Liu, Juncang Wu

**Affiliations:** ^1^ Department of Neurology, The Second People's Hospital of Hefei Hefei Hospital Affiliated to Anhui Medical University Hefei China; ^2^ Department of Neurology The Second People's Hospital of Hefei Affiliated to Bengbu Medical University Bengbu China; ^3^ Department of Neurology and Clinical Research Center of Neurological Disease the Second Affiliated Hospital of Soochow University Suzhou China; ^4^ Department of Neurology Anhui Medical University Hefei China

**Keywords:** cognitive function, nutritional status, Parkinson's disease, sarcopenia

## Abstract

**Purpose:**

This study aimed to investigate the association between sarcopenia and cognitive function in Chinese patients with Parkinson's disease (PD).

**Patients and methods:**

This cross‐sectional study included 148 patients with PD aged 60 years or older. Sarcopenia, nutritional status, and cognitive functioning were assessed using the 2019 Asian sarcopenia guidelines, the Mini Nutritional Assessment (MNA) scale, and the Montreal Cognitive Assessment (MoCA) scale, respectively. The relationship between sarcopenia and cognitive functioning was examined using multivariable logistic regression, receiver operating characteristic (ROC) analysis, and threshold effect analysis to identify the inflection point. Additionally, mediation analysis was performed to examine potential mediating variables. Subgroup analyses were also conducted to assess the robustness of the results across different populations.

**Results:**

In the fully adjusted model, higher MoCA scores were significantly associated with lower odds of sarcopenia (OR = 0.78, 95% CI: 0.69–0.88, *p* = 0.0001), whereas higher Hamilton Anxiety Inventory (HAMA) scores (OR = 1.11, 95% CI: 1.02–1.21, *p* = 0.0133) and Hamilton Depression Inventory (HAMD) scores (OR = 1.16, 95% CI: 1.06–1.27, *p* = 0.0011) were significantly associated with higher odds of sarcopenia. This association was more pronounced in older participants and those with higher HAMA scores. In addition, the multivariable model adjusted for age, gender, BMI, and MoCA showed better discriminative ability for sarcopenia than MoCA alone (AUC: 0.828 vs. 0.778). E‐value analysis suggested that the observed association was relatively robust to potential unmeasured confounding.

**Conclusion:**

Lower MoCA scores were independently associated with higher odds of sarcopenia in patients with PD. This association was more pronounced in older participants and those with higher anxiety scores. These findings suggest that cognitive assessment may help identify PD patients at increased likelihood of sarcopenia.

## Graphical abstract

Among 148 older patients with Parkinson's disease, lower MoCA scores were independently associated with higher odds of sarcopenia, while higher HAMA scores were also associated with sarcopenia. Cognitive assessment may help screen for sarcopenia risk in this population.

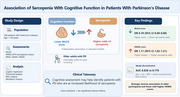

**Abbreviations**
ASM: appendicular skeletal mass
AWGS: Asian Working Group on Sarcopenia
BIA: bioelectrical impedance analysis
GS: gait speed
HAMA: Hamilton Anxiety Inventory
HAMD: Hamilton Depression Inventory
MoCA: Montreal Cognitive Assessment
PD: Parkinson's disease

## 1 Introduction

Sarcopenia is characterized by a progressive loss of skeletal muscle mass accompanied by a deterioration in muscular function (Marzetti et al. 2017; Beaudart et al. 2019), formally designated as a geriatric disease within the ICD‐10‐CM classification system. The 2019 update from the Asian Working Group on Sarcopenia (AWGS) establishes diagnostic criteria requiring evaluation of muscular mass, muscular power, and ambulatory velocity for sarcopenia identification (Chen, Woo, et al. 2020). In recent years, more and more studies have shown that single or complex factors such as muscle strength and mass are (Cawthon et al. 2020) the main causes of sarcopenia. Sarcopenia constitutes a primary contributor to disability, triggering detrimental sequelae including functional decline, recurrent inpatient admissions, and elevated all‐cause mortality risk (Sobestiansky et al. 2019; Leong et al. 2015). Studies have shown that 7%–10% of people aged 60–70 years and 30% of people over 80 years of age suffer from sarcopenia (Yan and Li 2024).

Cognitive impairment, a neurodegenerative process due to aging, involves functional impairments in several domains, including attention, memory, executive, language, computation, planning, and orientation (Ng et al. 2020). Aging plays an important role in both sarcopenia and cognitive impairment. Thus, sarcopenia and cognitive impairment share a common pathophysiologic pathway. The pathophysiological mechanisms of sarcopenia include aging, decreased activity, neuromuscular damage, insulin resistance, hormonal dysregulation, oxidative stress, and chronic inflammation (Hollingworth et al. 2021). In addition, these susceptibility conditions have been associated with cognitive impairment (Cruz‐Jentoft et al. 2010). It is not clear how sarcopenia affects cognitive function, but several studies have shown that a number of muscle factors are produced and secreted by skeletal muscle, including factors that regulate mood, learning, motor activity, and protection against neuronal damage, suggesting the presence of muscle‐brain communication (Scisciola et al. 2021). In addition, lifestyle factors, such as physical inactivity, poor diet, obesity, and smoking, are common risk factors for both disorders. On the other hand, sarcopenia may interact with cognitive function. Advanced sarcopenia and its accompanying fragility and loss of independence are clear causes of depression and low cognitive function (Knight and Durbin 2015). Conversely, cognitive impairment leads to reduced physical activity and dietary intake, which in turn accelerates the development of sarcopenia. There is now growing evidence that sarcopenia may be associated with an increased risk of cognitive impairment in older adults, although these findings are inconsistent (Lee et al. 2018; Abellan van Kan et al. 2013). A meta‐analysis confirmed a positive association between sarcopenia and cognitive impairment (Peng et al. 2020), but these results remain inconsistent in subgroup analyses by study population and study area.

Parkinson's disease (PD) is the second most common neurodegenerative disease in the world and is one of the fastest growing diseases in terms of morbidity, disability, and mortality (GBD 2016 Parkinson's Disease Collaborators 2018), and it is estimated that by 2030, the number of patients with PD in our country will reach 4.94 million, accounting for half of the global population with PD. The progression of PD is thought to be closely related to aging (Reeve et al. 2014). Previous studies by our team have shown that the prevalence of sarcopenia is significantly higher in PD patients than in the elderly (Q. Liu et al. 2023). On the other hand, cognitive impairment, as a unique non‐motor symptom in the clinical presentation of PD patients, may manifest at any stage in the disease progression of PD patients, and some of them may face cognitive impairment at the early stage of disease onset, which seriously affects the quality of life of PD patients.

There is an increasing number of studies on sarcopenia and cognitive function in the elderly, but there is a relative paucity of studies on sarcopenia and cognitive function in patients with PD. To improve the management and prognosis of patients with PD, it is important to identify factors associated with sarcopenia in this population.

## 2 Materials and Methods

### 2.1 Study Participants

**FIGURE 1:**
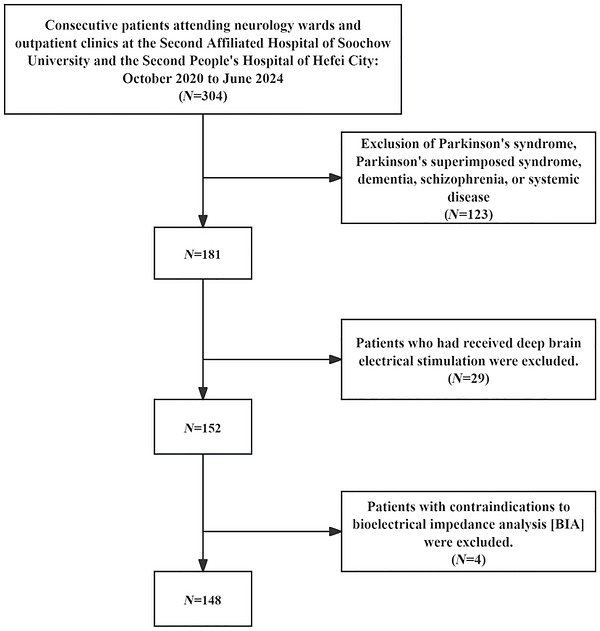
Flow chart of participants' selection.

We collected data from consecutive patients who visited the neurology ward and outpatient clinic of the Second Affiliated Hospital of Soochow University and the Second People's Hospital of Hefei between October 2020 and June 2024. Inclusion criteria included the following: (1) PD diagnosis according to the 2016 version of the Chinese PD diagnostic criteria. (2) Able to complete all scale measures. Exclusion criteria were as follows: (1) Parkinson's syndrome, Parkinson's superimposed syndrome caused by encephalitis, cerebrovascular disease, intoxication, trauma, and drugs. (2) Prior deep brain electrostimulation. (3) Dementia, schizophrenia, or systemic diseases. (4) Presence of contraindications to bioelectrical impedance analysis (BIA) (including implantation of medical devices). And the detailed screening process is shown in Figure 1.

Written informed consent was obtained from all enrolled participants. Ethical approval for this investigation was secured through the Institutional Review Boards of Soochow University Second Affiliated Hospital (Approval ID: LK‐2018‐061‐03) and Hefei Second People's Hospital (Protocol No.2025‐research‐093).

### 2.2 Data Collection

General information and clinical assessment: sex, age, education, body mass index (BMI), smoking status, history of alcohol consumption, tea or coffee consumption, and nutritional status were collected from all study participants. Education was classified according to the years of schooling, and BMI was calculated as weight divided by height in meters squared. Nutritional status was determined using the MNA scale, and patients were divided into two groups: those without risk of malnutrition (≥24 points) and those at risk of malnutrition (<24 points). The duration of the disease, medication, and comorbidities of patients with PD were recorded in detail. Anxiety and depressive symptoms were assessed using Hamilton Anxiety Inventory (HAMA) and Hamilton Depression Inventory (HAMD).

Standard neurologic evaluations are performed by board‐certified neurologists who thoroughly evaluate patients with PD during the medication onset phase.

### 2.3 Assessment and Diagnosis of Sarcopenia

Sarcopenia was diagnosed according to the 2019 AWGS consensus on the diagnosis and treatment of sarcopenia, which states that patients with reduced muscle strength or physical function and reduced muscle mass should be diagnosed with sarcopenia (Chen, Woo, et al. 2020).

#### 2.3.1 Muscle Strength

Camry hand dynamometer is used to measure hand grip strength (Guangdong Xiangshan Weighing Apparatus, Zhongshan City, Guangdong Province). The participant is asked to stand with both upper limbs hanging naturally at the sides of the body, then the maximum isometric strength is measured in the dominant hand or both hands, and the maximum reading is selected for recording after two tests. In men and women, low muscle strength was defined as < 28.0 and < 18.0 kg, respectively.

#### 2.3.2 Physical Function

The 6‐m gait speed(GS) test was used to assess physical function. Record the time taken to walk 6 m at a constant speed from the start of the exercise at a normal walking speed. Two tests were carried out, and the average of the two was used for the analysis. Reduced physical function was considered to be reflected by a GS ≤ 1m/s.

#### 2.3.3 Muscle Mass

Body composition was assessed using multifrequency BIA (InBody 720, Korea), which was considered in conjunction with body height to determine the appendicular skeletal mass (ASM) of each patient. After fasting and morning stool, all patients were assessed. Low muscle mass was defined as < 7.0 kg/m^2^ for men and < 5.7 kg/m^2^ for women.

### 2.4 Assessment of Cognitive Function

All study participants were assessed for cognitive function, and patients with PD were assessed 1–2 h after taking their medication. We used the Montreal Cognitive Assessment (MoCA) scale as a multidimensional neuropsychological instrument evaluating global cognitive capacity through eight domains: visuospatial‐executive competencies (5‐point scale), lexical retrieval (3‐point metric), immediate/delayed verbal recall (dual 5‐point indices), attentional regulation (6‐point continuum), linguistic processing (3‐point system), conceptual abstraction (2‐point framework), and spatiotemporal orientation (6‐point protocol) (Trzepacz et al. 2015). The MoCA has a composite value between 0 and 30, with higher composite values positively correlating with improved performance in cognitive functioning. In addition to age, education level has a strong influence on the variation of MoCA scores, which may adversely affect the test scores of individuals with low education. Our investigation implemented an educationally calibrated diagnostic framework for neurocognitive disorders, specifically tailored to the demographic characteristics (age strata and educational attainment) of the Han Chinese cohort under study. While extensive empirical evidence supports 23 as an established diagnostic threshold for cognitive deficits in cross‐cultural neuropsychological assessments (Carson et al. 2018; Luis et al. 2009; Saczynski et al. 2015), our investigation implemented a lenient classification framework given the unique demographic composition of the Han Chinese geriatric PD cohort (aged ≥ 60 years). Participants demonstrating MoCA composite scores below 22 met the criteria for neurocognitive impairment classification.

### 2.5 Statistical Analysis

All statistical analyses were performed using the Statistical Package for R version 4.1.3 (The R Foundation) and Empower software (X&Y solutions, Inc., Boston, MA, USA). Continuous variables demonstrating Gaussian distribution were presented using arithmetic mean with standard deviation (SD), whereas those exhibiting nonparametric characteristics were reported as median values with interquartile ranges (IQR), following initial normality verification through Kolmogorov–Smirnov testing. An initial assessment of normality via the Kolmogorov–Smirnov test revealed a non‐normal distribution of data. For non‐normally distributed continuous variables, the median and tertiles were used to describe the data, and the Kruskal–Wallis H‐test was used for comparisons between groups. Categorical data were presented using proportions, with the chi‐square test applied for categorical variables. Subsequently, we used multivariate logistic regression analyses and generalized additive models (GAMs) to investigate the relationship between cognitive impairment and sarcopenia in patients with PD and its nonlinearity. We categorized MoCA scores into tertiles to assess the relationship between patients with high, medium, and low‐tertile scores and sarcopenia, which was examined by restricted cubic spline function (RCS) regression. Subgroup analyses were also performed for age, HAMA, and HAMD. Mediation effect analyses were conducted for gender, MNA classification, and HAMD and HAMA scores to reveal the underlying possible mechanisms. To evaluate the discriminative ability of MoCA for sarcopenia in patients with PD, receiver operating characteristic (ROC) curve analysis was performed, and the area under the curve (AUC) was calculated using the C‐statistic. Additionally, we explored the effect of possible unmeasured confounders between the MoCA score and sarcopenia by calculating E‐values. The E‐value quantifies the magnitude of unmeasured confounders that may be required to counteract the observed association between MoCA score and sarcopenia (VanderWeele and Ding 2017). All statistical tests were two‐tailed, and a *p*‐value < 0.05 was considered statistically significant.

## 3 Results

### 3.1 General Information on the Study Population

**FIGURE 2:**
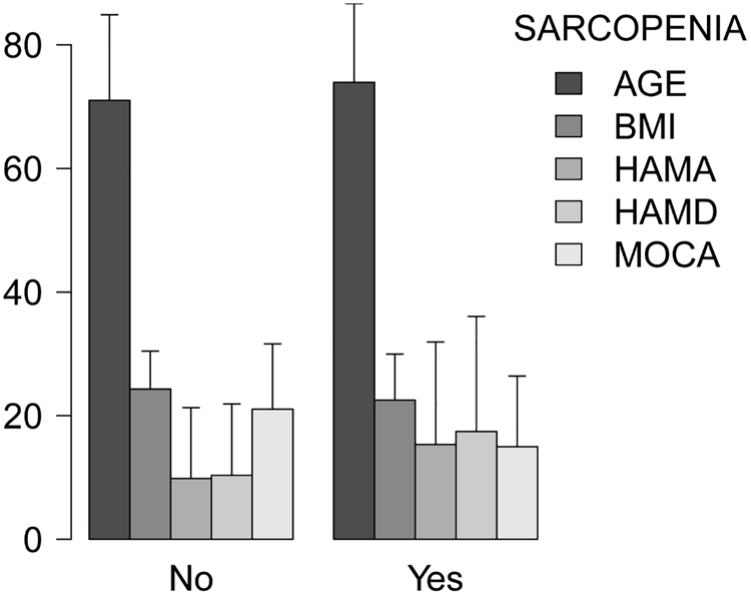
Baseline composite bar plots of populations for selected variables.

This study included 148 patients (76 males and 72 females) with PD, with a mean age of 72.37 years and a median of 6 years of education (Table 1). According to the 2019 AWGS criteria, 69 individuals were eligible for sarcopenia, with a prevalence of 46.62%. After stratifying MoCA scores into tertiles, we found that muscle mass, grip strength, and BMI increased with higher MoCA scores, whereas HAMA and HAMD scores decreased (Table 1). Figure 2 shows that, compared with patients without sarcopenia, those with sarcopenia had broadly similar age and BMI but lower total MoCA scores and higher HAMA and HAMD scores.

**TABLE 1:** Basic characteristics of participants with Parkinson's MoCA scores in China.

MOCA score tertile	Low	Middle	High	*p*‐value
*N*	42	52	54	
Age	74.14 ± 7.04	72.56 ± 6.84	70.81 ± 6.74	0.063
BMI	22.77 ± 3.41	23.58 ± 3.86	23.88 ± 3.37	0.308
LED	426.20 ± 304.08	393.01 ± 368.31	483.24 ± 289.08	0.350
Muscle mass	5.88 ± 0.97	6.58 ± 1.02	7.00 ± 1.17	< 0.001
Physical function	9.83 ± 2.96	8.36 ± 2.88	8.38 ± 2.19	0.013
Muscle strength	18.92 ± 8.81	20.81 ± 8.49	24.76 ± 8.52	0.003
HAMA	16.14 ± 8.88	11.19 ± 7.15	10.61 ± 6.13	< 0.001
HAMD	17.33 ± 9.45	12.62 ± 8.71	11.80 ± 6.66	0.003
Gender				0.831
Male	20 (47.62%)	28 (53.85%)	28 (51.85%)	
Female	22 (52.38%)	24 (46.15%)	26 (48.15%)	
Educational attainment (years)				0.006
< 12	41 (97.62%)	49 (94.23%)	43 (79.63%)	
≥ 12	1 (2.38%)	3 (5.77%)	11 (20.37%)	
Smoke				0.052
No	39 (92.86%)	40 (76.92%)	40 (74.07%)	
Yes	3 (7.14%)	12 (23.08%)	14 (25.93%)	
Tea or coffee				< 0.001
No	40 (95.24%)	42 (80.77%)	33 (61.11%)	
Yes	2 (4.76%)	10 (19.23%)	21 (38.89%)	
Alcohol				0.194
No	36 (85.71%)	47 (90.38%)	42 (77.78%)	
Yes	6 (14.29%)	5 (9.62%)	12 (22.22%)	
MNA classification				< 0.001
No Malnutrition	4 (9.52%)	20 (38.46%)	40 (74.07%)	
Undernutrition	38 (90.48%)	32 (61.54%)	14 (25.93%)	
Hypertension				0.227
No	22 (52.38%)	36 (69.23%)	35 (64.81%)	
Yes	20 (47.62%)	16 (30.77%)	19 (35.19%)	
Diabetes				0.714
No	37 (88.10%)	47 (90.38%)	46 (85.19%)	
Yes	5 (11.90%)	5 (9.62%)	8 (14.81%)	
Sarcopenia				< 0.001
No	9 (21.43%)	27 (51.92%)	43 (79.63%)	
Yes	33 (78.57%)	25 (48.08%)	11 (20.37%)	

*Note*: Table results: Mean + SD/*N* (%). *p*‐value: Kruskal–Wallis rank sum test for continuous variables, and Fisher's exact probability test for count variables with theoretical number < 10. MoCA score: T1 ≤ 14,14 < T2 < 22, T3 ≥ 22.

### 3.2 The Relationship Between Cognitive Impairment and Sarcopenia in Patients With PD

**FIGURE 3:**
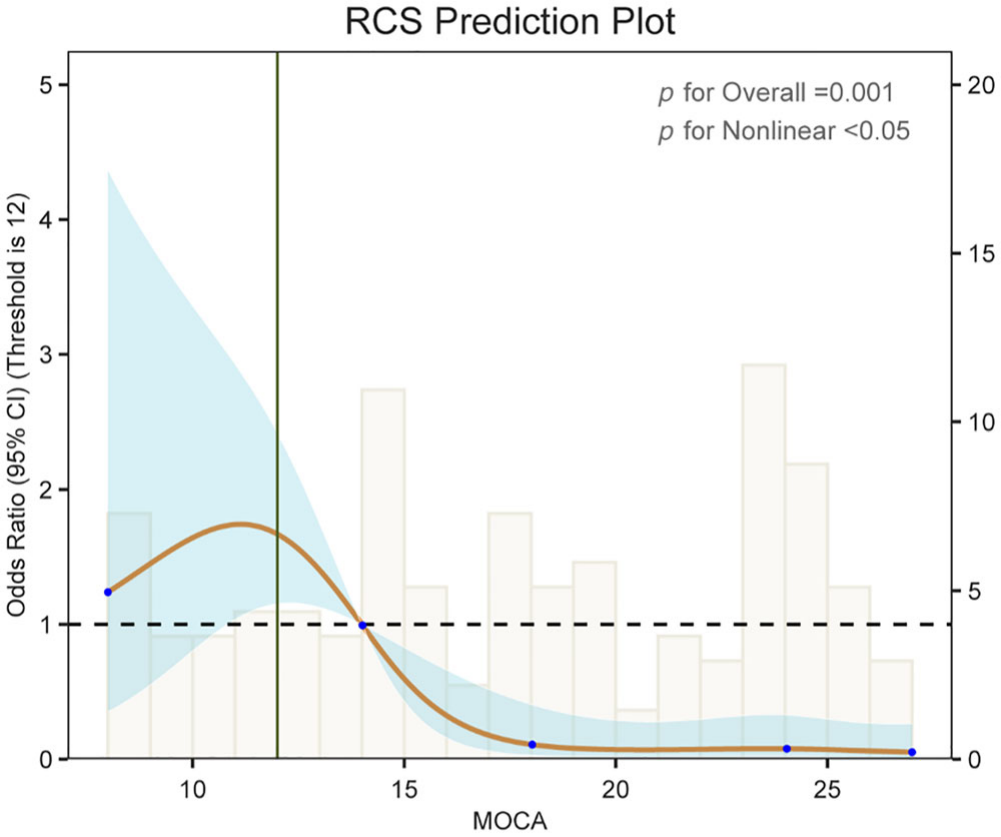
The RCS curve of the association between MoCA and sarcopenia among all the study participants. The curves represent restricted cubic splines between variables, blue shading represents 95% confidence intervals for the fitted results, and the solid green line represents the threshold. A nonlinear relationship between MoCA score and sarcopenia was detected by generalized additive modeling.

Table 2 shows that higher MoCA scores were consistently associated with lower odds of sarcopenia across all three models, whereas higher HAMA and HAMD scores were associated with higher odds of sarcopenia. After adjusting for all confounding variables, the odds of sarcopenia decreased by 22% for each one‐unit increase in MoCA score in Model 3 (OR = 0.78, 95% CI: 0.69, 0.88). In contrast, the odds of sarcopenia increased by 11% (OR = 1.11, 95% CI: 1.02, 1.21) and 16% (OR = 1.16, 95% CI: 1.06, 1.27) for each one‐unit increase in HAMA and HAMD scores in Model 3. When patients with PD were stratified by MoCA tertiles, those in the highest tertile (T3) had substantially lower odds of sarcopenia than those in the lowest tertile (T1). The RCS plot showed an inverse association between MoCA score and sarcopenia (Figure 3). Threshold effect analysis identified an inflection point at a MoCA score of 12. On the left side of this inflection point, the association was not statistically significant (OR = 1.17, 95% CI: 0.83–1.64), whereas on the right side, higher MoCA scores were significantly associated with lower odds of sarcopenia (OR = 0.70, 95% CI: 0.59–0.83). E‐value quantification was implemented to assess the robustness of observed covariation between MoCA metrics and sarcopenic pathology against potential unobserved confounders. The results of the E‐value analyses showed that unmeasured confounders needed to have an association strength of 1.88 with exposure (MoCA score) and outcome (sarcopenia) to fully explain the observed associations. In addition, the observed associations may be weakened or strengthened if the strength of association of the unmeasured confounders with exposure is lower than 1.54 or with outcome is higher than 2.68.

**TABLE 2:** Association between MoCA and sarcopenia.

Exposure	Non‐adjusted	Adjust I	Adjust II
MoCA	0.83 (0.78, 0.89) < 0.0001	0.76 (0.68, 0.86) < 0.0001	0.78 (0.69, 0.88) 0.0001
HAMA	1.12 (1.06, 1.18) < 0.0001	1.12 (1.04, 1.21) 0.0026	1.11 (1.02, 1.21) 0.0133
HAMD	1.13 (1.07, 1.19) < 0.0001	1.16 (1.07, 1.26) 0.0002	1.16 (1.06, 1.27) 0.0011
MoCA tertile			
T1: Low	1.0	1.0	1.0
T2: Middle	0.25 (0.10, 0.63) 0.0032	0.18 (0.05, 0.65) 0.0085	0.18 (0.04, 0.73) 0.0165
T3: High	0.07 (0.03, 0.19) < 0.0001	0.02 (0.00, 0.09) < 0.0001	0.02 (0.00, 0.15) 0.0001

*Note*: Non‐adjusted model: no covariates were adjusted for. Adjust I model: adjusted for sex, age, education, body mass index (BMI), smoking, tea or coffee consumption, and alcohol consumption. Adjust II model: adjusted for sex, age, education, body mass index (BMI), smoking, tea or coffee consumption, alcohol consumption, Mini Nutritional Assessment (MNA), hypertension, diabetes, and levodopa equivalent dose (LED).

### 3.3 Subgroup Analyses, Mediation Analyses, and ROC Analysis of the Relationship Between MoCA Scores and Sarcopenia

**FIGURE 4:**
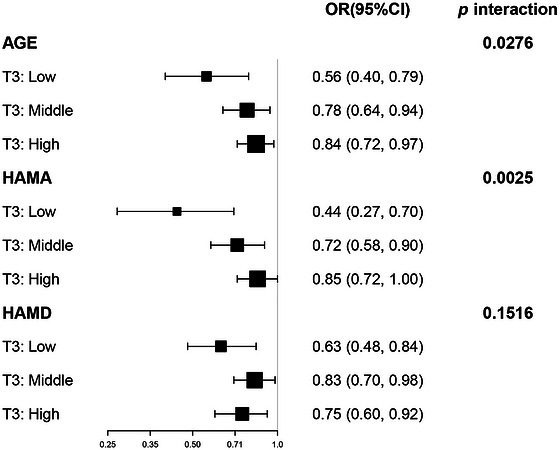
Subgroup analysis of the relationship between MoCA and sarcopenia. The model was adjusted for age, gender, years of education, smoking, alcohol, tea or coffee consumption, LED values, BMI, hypertension, and diabetes.

**FIGURE 5:**
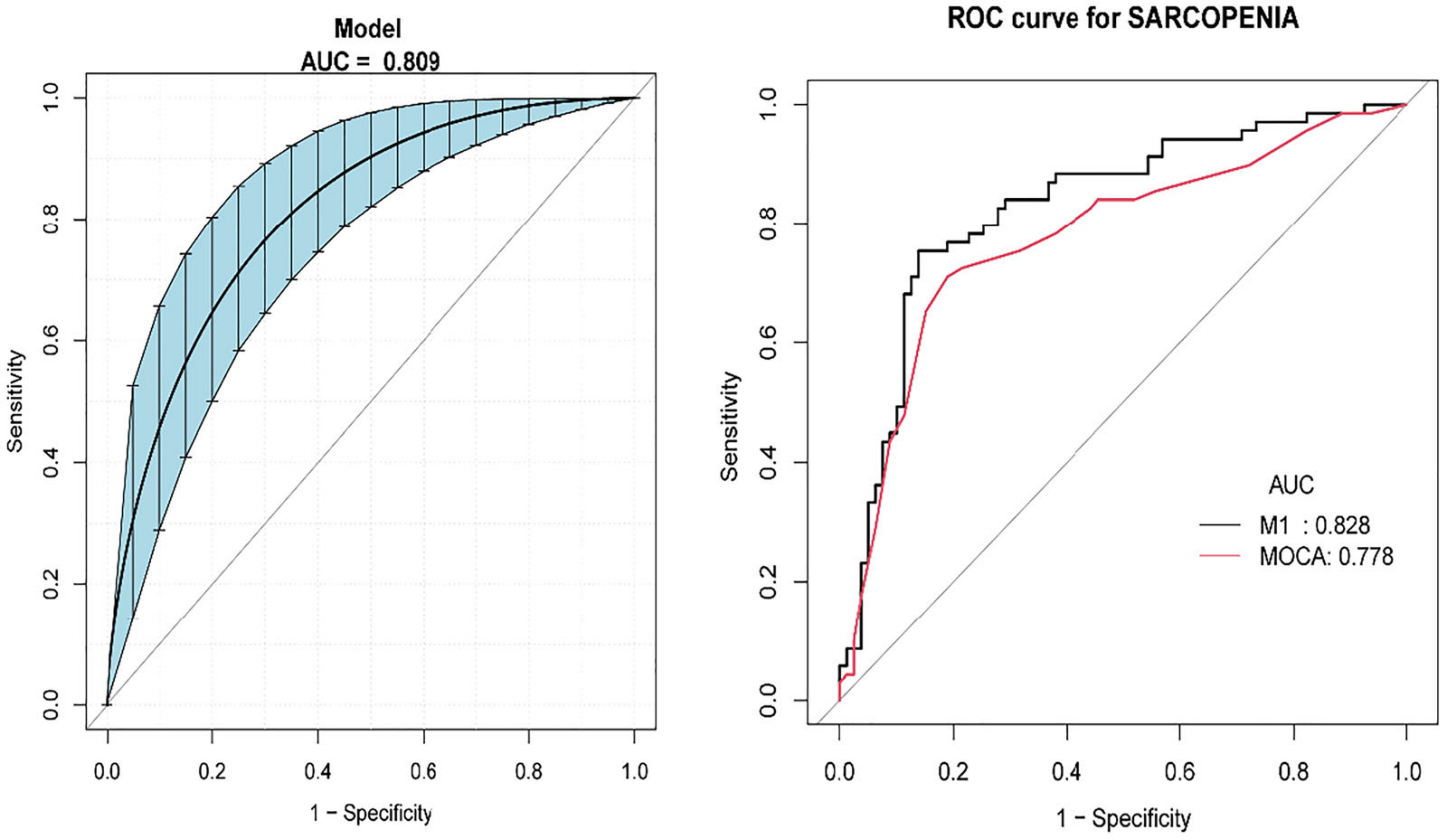
Comparison of ROC curves for sarcopenia between models. M1 model adjusted for age, gender, BMI, and MoCA. Blue shading shows the bootstrap estimated 95% CI with the AUC.

Subgroup analyses were performed according to age, HAMA score, and HAMD score. As shown in Figure 4, no significant interaction was observed for HAMD score (*p* > 0.05), whereas significant interactions were observed for age and HAMA score (both *p* < 0.05). The inverse association between MoCA score and sarcopenia was more pronounced in older participants and those with higher HAMA scores. In addition, Figure 5 shows the results of the ROC curve analysis. The M1 model (adjusted for age, gender, BMI, and MoCA) yielded an AUC of 0.828, indicating better discriminative performance for sarcopenia than MoCA alone (AUC = 0.778). In addition, we analyzed mediating effects for HAMA, HAMD, gender, and MNA. In addition, mediation analyses were performed for HAMA score, HAMD score, gender, and MNA classification. As shown in Table 3, no significant indirect effects were identified for any of these variables (Table 3).

**TABLE 3:** Analysis of the mediating effect of MoCA and sarcopenia.

	Mediation effect (95% CI)	Direct effect (95% CI)	Proportion mediated (95% CI)
Gender	−0.03 (−0.08, 0.01)	−0.36 (−0.51, −0.20)	0.07 (−0.02, 0.20)
HAMD	0.01 (−0.05, 0.06)	−0.33 (−0.49, −0.18)	−0.02 (−0.29, 0.16)
MNA classification	−0.06 (−0.16, 0.01)	−0.18 (−0.49, 0.17)	0.16 (0.03, 0.43)
HAMA	−0.02 (−0.06, 0.01)	−0.06 (−0.47, 0.13)	0.05 (0.03, 0.23)

*Note*: Adjusting variables: education, smoke, alcohol, tea or coffee, gender, age, MNA, hypertension, diabetes, BMI, and LED.

## 4 Discussion

This study demonstrated a significant negative association between MoCA scores and sarcopenia in Chinese patients with PD. Better cognitive function was associated with lower odds of sarcopenia, and this association remained robust after adjustment for potential confounders. Cognitive impairment was closely and independently correlated with sarcopenia in PD patients.

Previous studies on sarcopenia and cognitive function have yielded inconsistent results (Jang and Kim 2015; X. Liu et al. 2020; Nishiguchi et al. 2016; Burns et al. 2010; Hsu et al. 2014), which may be attributed to differences in cognitive assessment tools and study populations. Most studies were conducted in the general elderly population, whereas evidence in patients with PD remains scarce. The MoCA has good sensitivity for detecting early cognitive impairment in patients with PD (Wood et al. 2020). In the present study, we measured muscle mass using BIA and found that MoCA scores were significantly lower in PD patients with sarcopenia. This may be explained by the fact that BIA is more suitable for elderly individuals, and the association between body composition and cognitive impairment identified by BIA may be stronger than that detected by dual‐energy x‐ray absorptiometry (DXA) (Won et al. 2017; Kim and Won 2019).

There are several potential biological links between sarcopenia and cognitive decline. First, cognitive decline reduces physical activity and food ingestion, which can trigger excessive muscle loss and accelerate sarcopenia in older adults (Roubenoff 2002). Second, chronic inflammation can mediate muscle loss and cognitive dysfunction. Higher levels of Interleukin‐6 are associated with the loss of skeletal muscle mass (Dalle et al. 2017) and an increased risk of dementia (Kimura et al. 2019). In addition, excessive oxidative stress associated with chronic diseases plays an essential role in muscle atrophy by disrupting the balance between protein synthesis and catabolism, causing mitochondrial dysfunction and inducing apoptosis, leading to sarcopenia (Gonzalez et al. 2021). In contrast, a major risk factor for cognitive impairment is the accumulation of oxidative and nitrosative stress products with age (Franzoni et al. 2021). Malnutrition is also a common risk factor, and nutritional status has been reported to mediate the relationship between sarcopenia and cognitive impairment (Hu et al. 2021).

Furthermore, unlike studies in the general elderly population, our study focused on the unique pathophysiology of PD. PD is characterized by progressive loss of dopaminergic neurons in the substantia nigra and widespread propagation of α‑synuclein‑positive Lewy bodies (Weintraub et al. 2022). These core lesions interact with common pathways of sarcopenia and cognitive impairment, including chronic inflammation and oxidative stress (Dalle et al. 2017; Gonzalez et al. 2021). Dopaminergic depletion impairs central regulation of muscle metabolism and promotes muscle atrophy, while Lewy body spread drives cognitive deterioration (Weintraub et al. 2022). Inflammation and oxidative stress, as key drivers of PD pathogenesis, simultaneously accelerate muscle loss and neuronal damage (Dalle et al. 2017; Gonzalez et al. 2021). Thus, PD‑specific pathology forms a vicious cycle with sarcopenia and cognitive impairment, which explains the higher prevalence of both disorders in PD patients than in the general elderly population.

Subgroup analyses showed that the inverse association between MoCA score and sarcopenia was stronger in older patients and those with higher HAMA scores, indicating that age and anxiety may be important effect modifiers. With advancing age, neurodegeneration and muscle loss progress in parallel, amplifying the link between cognitive impairment and sarcopenia. Anxiety activates the HPA axis and increases cortisol secretion, which damages the hippocampus and disrupts muscle perfusion and metabolism, thereby exacerbating both cognitive dysfunction and sarcopenia (Vreeburg et al. 2010; Mantella et al. 2008).

In the present study, mediation analyses did not identify significant indirect effects of gender, HAMD score, MNA classification, or HAMA score on the association between MoCA scores and sarcopenia. This negative finding deserves attention, as previous studies have suggested that nutritional status, mood symptoms, and demographic characteristics may play mediating roles in the association between muscle health and cognitive function (Hu et al. 2021; Chen, Hwang, et al. 2020). Several potential reasons may explain this result. First, the relatively small sample size may lead to insufficient statistical power, increasing the risk of type II error and failing to detect weak or moderate mediating effects. Second, the assessment tools in this study were relatively single, including only one scale for anxiety, depression, and nutritional status, which may limit the sensitivity and comprehensiveness of measurement, resulting in measurement bias and failing to capture the complete mediating pathway. Third, it may be that these variables do not act as independent mediators in PD patients, and the association between cognitive impairment and sarcopenia may be driven by other unmeasured factors such as chronic inflammation, oxidative stress, physical activity and other pathophysiological mechanisms, which were not included in the mediation model. In the future, studies with larger sample sizes, more comprehensive evaluation tools and more comprehensive confounding factor adjustment are needed to further explore whether other variables play a mediating role in the relationship between cognitive function and sarcopenia in PD patients.

Our study has several strengths. It is the first to explore the association between sarcopenia and cognitive function in Chinese PD patients using BIA and MoCA. E‐value analysis confirmed the robustness of the results. However, several limitations should be noted. First, the cross‐sectional design precludes causal inference. Future prospective cohort studies are needed to clarify the causal pathways and pathological mechanisms underlying sarcopenia and cognitive decline in PD. Second, the sample size was relatively small, and PD subtypes were not distinguished. Third, cognitive function was assessed solely by the MoCA, which may not comprehensively evaluate all cognitive domains. Accordingly, more extensive neuropsychological test batteries should be applied in future research.

## 5 Conclusion

In conclusion, our study demonstrates a negative association between MoCA scores and sarcopenia in Parkinson's patients, and it is more pronounced in the advanced age and high HAMA score groups. Our findings suggest that straightforward questionnaires and demographic factors may help clinicians identify patients with PD who are more likely to have sarcopenia and may therefore warrant further assessment. Researchers are encouraged to further investigate the relationship between cognitive impairment and sarcopenia in future geriatric care and management strategies. Whether interventions targeting sarcopenia can improve cognitive outcomes in patients with PD remains to be determined in future prospective studies. Such interventions could facilitate the early detection of sarcopenia and cognitive impairment, potentially slowing disease progression, improving quality of life, and alleviating the social and economic burdens faced by older adults.

## Author Contributions

**Kangrui Zhang**: conceptualization, methodology. **Qiuwan Liu**: conceptualization, methodology. **Juncang Wu**: conceptualization, methodology, funding acquisition, writing – review and editing. **Yi Tang**: writing – original draft, conceptualization, methodology. **Chi Zhang**: investigation. **Chunfeng Liu**: conceptualization, methodology. **Chengjie Mao**: methodology, conceptualization.

## Funding

This work was supported by the Jiangsu Provincial Medical Key Discipline (ZDXK202217), Jiangsu Provincial Key R&D Program (BE2018658), Hefei key common technology research and development project (GJ2022M07), Natural Science Key Program of Bengbu Medical University (2024byzd398), and Natural Science Key Program of Bengbu Medical University (2024byzd401).

## Ethics Statement

This study was approved by the Ethics Review Committees of the Second Affiliated Hospital of Soochow University (approval number: LK‐2018‐061‐03) and the Second People's Hospital of Hefei (NO. 2025‐research‐093). All methods were performed in accordance with relevant guidelines and regulations. All participants provided written informed consent.

## Conflicts of Interest

The authors declare no conflicts of interest.

## Data Availability

The datasets generated and analyzed during the current study are not publicly available due to privacy and ethical restrictions but are available from the corresponding author on reasonable request.
